# Linking Cascading Effects of Fish Predation and Zooplankton Grazing to Reduced Cyanobacterial Biomass and Toxin Levels Following Biomanipulation

**DOI:** 10.1371/journal.pone.0112956

**Published:** 2014-11-19

**Authors:** Mattias K. Ekvall, Pablo Urrutia-Cordero, Lars-Anders Hansson

**Affiliations:** 1 Lund University, Department of Biology, Ecology Building, SE-223 62, Lund, Sweden; 2 Lund University, Center for Environmental and Climate Research, Ecology Building, SE-223 62, Lund, Sweden; University of Connecticut, United States of America

## Abstract

Eutrophication has been one of the largest environmental problems in aquatic ecosystems during the past decades, leading to dense, and often toxic, cyanobacterial blooms. In a way to counteract these problems many lakes have been subject to restoration through biomanipulation. Here we combine 13 years of monitoring data with experimental assessment of grazing efficiency of a naturally occurring zooplankton community and a, from a human perspective, desired community of large *Daphnia* to assess the effects of an altered trophic cascade associated with biomanipulation. Lake monitoring data show that the relative proportion of *Daphnia* spp. grazers in June has increased following years of biomanipulation and that this increase coincides with a drop in cyanobacterial biomass and lowered microcystin concentrations compared to before the biomanipulation. In June, the proportion of *Daphnia* spp. (on a biomass basis) went from around 3% in 2005 (the first year of biomanipulation) up to around 58% in 2012. During months when the proportion of *Daphnia* spp. remained unchanged (July and August) no effect on lower trophic levels was observed. Our field grazing experiment revealed that *Daphnia* were more efficient in controlling the standing biomass of cyanobacteria, as grazing by the natural zooplankton community never even compensated for the algal growth during the experiment and sometimes even promoted cyanobacterial growth. Furthermore, although the total cyanobacterial toxin levels remained unaffected by both grazer communities in the experimental study, the *Daphnia* dominated community promoted the transfer of toxins to the extracellular, dissolved phase, likely through feeding on cyanobacteria. Our results show that biomanipulation by fish removal is a useful tool for lake management, leading to a top-down mediated trophic cascade, through alterations in the grazer community, to reduced cyanobacterial biomass and lowered cyanobacterial toxin levels. This improved water quality enhances both the ecological and societal value of lakes as units for ecosystem services.

## Introduction

Human impact on aquatic ecosystems, in particular in the form of eutrophication, has been one of the largest environmental problems for aquatic ecosystems during the past decades [1], [2]. The subsidy of nutrients, and specifically phosphorus [3], [4], to inland waters from the surrounding landscapes lead to increased primary production and facilitates the formation of cyanobacterial blooms during warm summer months [5]. These cyanobacterial blooms, often dominated by different cyanobacterial genera such as *Microcystis spp. and Anabaena spp*., often have the potential to produce a vast array of toxins, such as microcystins and anatoxin-a [6]–[9], posing a serious threat to both humans [10], [11] and aquatic organisms [12]. Furthermore, cyanobacterial blooms can have considerable impact on both biodiversity and ecosystem functioning, as well as ecosystem services, such as recreation and drinking water supply, making the water resource less desirable [13].

As a counter-action to those alterations, researchers and stakeholders have invested considerable efforts in finding ways to restore reservoirs and lakes to their former glory. Several restoration measures, both in the catchments and in the lakes themselves, have been proposed. Measures in the catchment mainly focus on reducing the external nutrient loading to the lakes, for example by constructing buffer zones around streams in agricultural areas, reducing point sources, and constructing wetlands to catch the nutrients before reaching the lake [14]. In-lake measures include for example chemical treatment of the sediment to reduce internal nutrient loading [15] and different kinds of biomanipulations [16]. Biomanipulations, a term first coined by Shapiro et al. [17], can be of many different types including cyprinid fish removal, as well as zooplankton and piscivorous fish stocking or a combination of both [18]–[22]. Regardless of biomanipulation type, they all have the common goal of altering the food web and ultimately increase the grazing pressure on phytoplankton, thereby reducing the occurrence of algal blooms through top-down effects [21], [23]. Due to high fish predation, the zooplankton communities in eutrophic lakes are generally dominated by small sized species which are less effective in feeding on large phytoplankton compared to large cladoceran zooplankton species, such as *Daphnia magna* [24]. This means that although there might be relatively high numbers of zooplankton, the existing species composition is rarely favoring grazing on the targeted large cyanobacteria, thereby facilitating bloom formation.

In this study we address the question whether alterations in the food chain composition, such as a biomanipulation, can improve the water quality with respect to cyanobacterial biomass and toxin production. To address this we used 13 years of monitoring data from the eutrophic Lake Ringsjön, southern Sweden, of which the last eight years have been subject to cyprinid fish removal (biomanipulation) to track the trophic cascade through the food web. In addition, we performed a field grazing experiment to simulate how an increased abundance of large cladoceran grazers, *Daphnia magna*, would affect grazing efficiency on the phytoplankton community compared to the naturally occurring zooplankton community dominated by small taxa. We hypothesized that an increased amount of large cladoceran grazers would increase the grazing pressure on cyanobacteria relative to the existing zooplankton community and thereby alter the amount of cyanobacterial toxins in the lake.

## Materials and Methods

### Site description

Lake Ringsjön is a eutrophic lake situated in the southern part of Sweden. The lake consists of three connected basins with a total area of 40 km^2^ and has been subject to biomanipulation by cyprinid fish removal, mainly roach (*Rutilus rutilus*) and bream (*Abramis brama*) [25]. The first biomanipulation attempt in Lake Ringsjön was between 1989–1992 [16] and the effects of this biomanipulation lasted until the mid 1990s, when the lake showed signs to return to a turbid state. As a response to the increased turbidity, a new attempt of biomanipulation was initiated in 2005 and was still ongoing at the time of the field study in 2012. Here we focus on this latest effort of biomanipulation in the Western basin of Lake Ringsjön (area: 14.8 km^2^, max depth 5.4 m) using available monitoring data from 2000–2004 as a reference for evaluating the changes in the lake during the eight years with biomanipulation (2005–2012).

### Lake monitoring data

Lake Ringsjön has been subject to monthly monitoring of water chemistry (total phosphorous), total chlorophyll *a* concentration and phytoplankton- and zooplankton biomass for more than 10 years. Moreover, since 2004 samples for cyanobacterial toxins, specifically microcystins, have been taken on a monthly to bi-weekly basis. The microcystin samples were taken in surface water above the deep-hole of the western basin and were immediately frozen and later analyzed using enzyme-linked immunoassay (Microcystins-DM ELISA Microtiter Plate, Abraxis LLC, Warminster, PA, USA) according to Hansson et al. (2007) [12]. In addition, as a proxy for cyprinid fish abundance, the landings of cyprinid fish from the trawling during the biomanipulation were used to assess the trends in the targeted fish stock. The same type and number of trawlers were used during all years of biomanipulation. As the number of days trawled differed slightly between years due to e.g. weather conditions, catches were normalized to trawling effort and, besides total catch, catch efficiency (i.e. catch per trawling day) were estimated for each year. Predatory fish (mainly pike (*Esox lucius*), pikeperch (*Sander lucioperca*) and large perch (*Perca fluviatilis*)) caught in the trawl were immediately sorted out on the trawling boat and returned to the lake and is hence not included in the landings.

### Field grazing-experiment

A field experiment, based on the method described by Lehman and Sandgren (1985) [26], consisting of 12 plastic cubitainers (volume: 10 L) was run once a month between June and August 2012. Cubitainers were divided into two gradients (n = 6) to investigate two different outcomes of lake biomanipulations. The first gradient consisted of a biomass gradient of the natural zooplankton community that exists in the lake today, while the second gradient constituted a gradient representing a scenario where a large cladoceran grazer, here represented by *Daphnia magna*, becomes the dominant herbivore in the lake. Hence, this gradient was designed to represent a future scenario where the fish predation had been reduced to very low levels. The *Daphnia* used were taken from a lab-reared population. These *Daphnia* had been fed with a mixture of phytoplankton during several generations consisting mainly of green algae but also containing low levels of cryptophytes and cyanobacteria.

**Figure 1:**
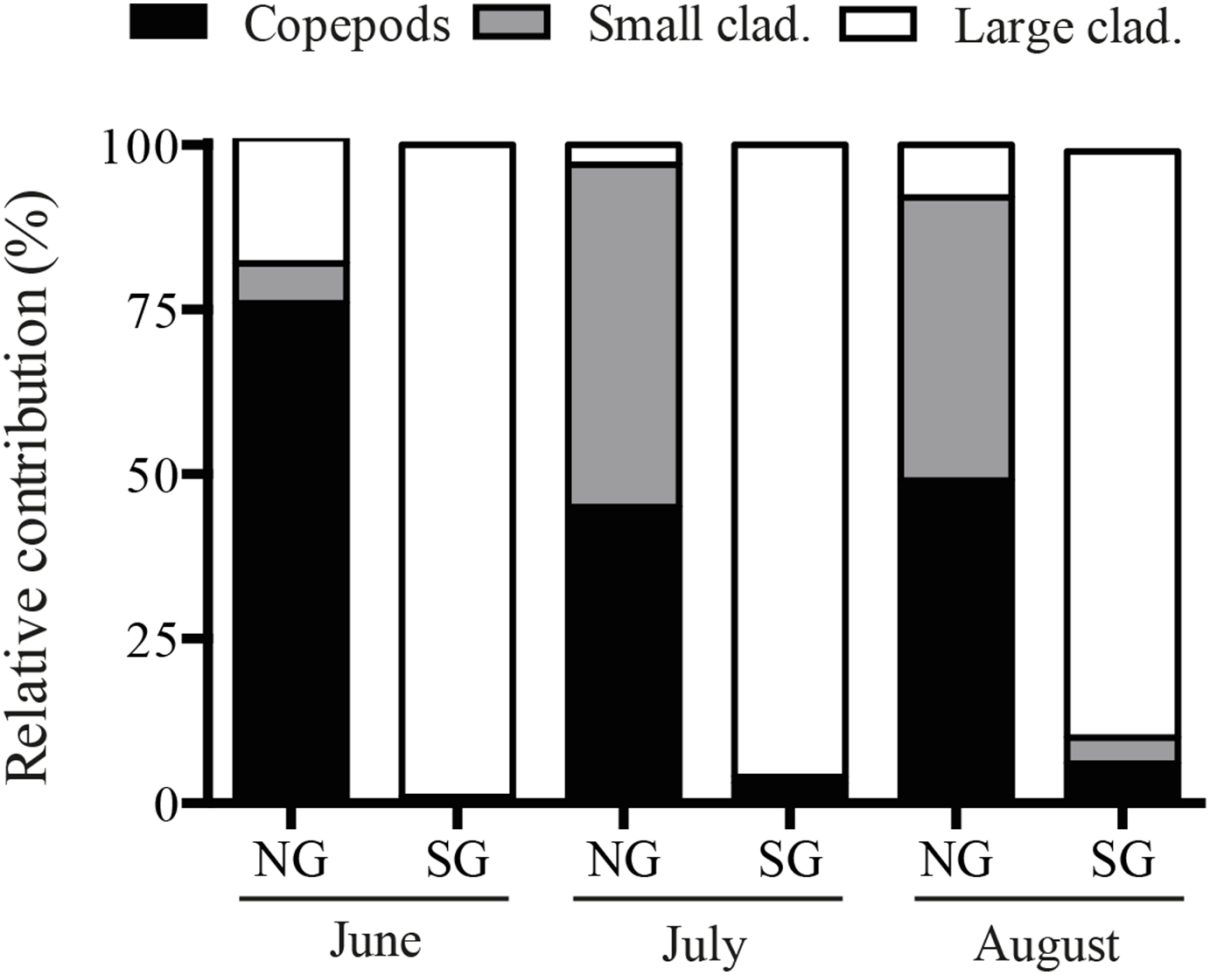
Relative community composition (%), on a biomass basis, of copepods, small cladocerans and large cladocerans in the different grazer communities in June, July and August, respectively. NG = naturally occurring grazer community, SG = standardized (*Daphnia* dominated) grazer community.

Each cubitainer received 9 L of 150-µm filtered lake water i.e. water containing the natural phytoplankton community without grazers larger than 150 µm. The zooplankton retrieved on the 150-µm filter were pooled and subsequently added to the cubitainers constituting the natural-grazer gradient in 0.25, 0.5, 1, 2, 4 and 6 times the biomass of zooplankton in the lake at the time of sampling (for absolute biomasses see result section). This grazer community was dominated by copepods and small cladocerans (Chydorids, *Bosmina* spp. and *Ceriodaphnia* spp.), the latter defined as smaller than 400 µm in length. The second gradient received a gradient of cultured *Daphnia magna* (mean ±SD size: 1750.9±376.8 µm) following the same gradient steps as for the natural grazers with 8±4 *Daphnia magna* per liter (mean±SD during the different months) in the “ambient step” (1) depending on the size of the individuals in the culture, thereby successfully generating two different grazer community compositions (Fig. 1). Although these gradients differed in their absolute biomass, with higher total biomasses in the *Daphnia*-dominated gradients due to the large size of *Daphnia magna* and the small size of the individuals in the natural community, the natural grazers always outnumbered the *Daphnia*-gradient. Once filled, 0-samples were taken for phytoplankton enumeration, total microcystin concentrations and extracellular microcystin concentrations. Thereafter the cubitainers were hooked onto a rope and incubated in the lake for 72 hours in the surface water. The cubitainers were then taken out of the lake and samples were taken for phytoplankton and microcystin concentrations. Also, the entire volume in the cubitainer was filtered through a 150-µm mesh and the zooplankton retrieved on the filter were kept as a measure for grazer community composition and biomass determination. Samples for extracellular microcystins were collected by, prior to freezing, removing all cyanobacterial cells from the water by a very low-pressure filtration using GF/C-filters (Whatman). All microcystin samples were stored at −20°C until analyzed in the same way as the lake monitoring samples with the exception that extracellular samples, which contained no cells, were not sonicated. All plankton samples were fixed with Lugoĺs solution and stored in a cooling room at 4°C for later enumeration and biomass determination. Zooplankton samples were counted and measured using a stereoscopic microscope (Olympus SZ40) at 20x magnification and biomasses were estimated using length-weight regressions according to Bottrell et al. [27] and Dumont et al. [28]. Cyanobacteria were counted using an inverted microscope (Olympus CK40) and biomasses were estimated according to the methods described by Ekvall et al. (2013) [6], whereafter net cyanobacterial growth rates (r) were calculated using the equation: r = ln(N_t_/N_0_)/Δt, where N_t_ is the final cyanobacterial biomass, N_0_ the initial cyanobacterial biomass and Δt the running time of the experiment. Calculated growth rates were then correlated to grazer abundance and grazing rates were estimated based on Lehman and Sandgren (1985) [26]. No specific permits were needed to conduct this experiment.

### Data analysis and statistics

All lake data were split into either before biomanipulation (2000–2004) or during biomanipulation (2006–2012) and were analyzed on a monthly basis using linear regressions with year as independent variable and the respective response variable as dependent variable. The first year of biomanipulation (2005) were considered a transition year and were not included in any of the two groups. As only one data point on microcystins exists prior to the biomanipulation (2004) changes in microcystin concentration were only analyzed for years with biomanipulation (i.e. changes over time with years of ongoing biomanipulation). The results of the grazer-gradient experiment were also analyzed via linear regressions using net growth rate (r) as dependent- and zooplankton biomass as independent variable. The same approach was used for both the total microcystin concentration, as well as the extracellular concentration although the r in this case represented changes in toxin concentration rather than growth rate. Statistical analyses were made in SPSS 21 for Macintosh (grazing experiment) and Prism 6 for Macintosh (lake monitoring data).

## Results

### Lake monitoring

**Figure 2:**
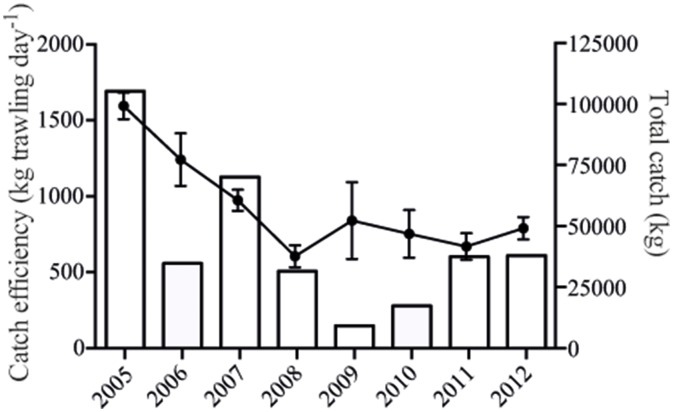
Total reported landings from the trawl per year (bars) and average catch efficiency (kg trawling day^−1^) ± SE (line).

**Figure 3:**
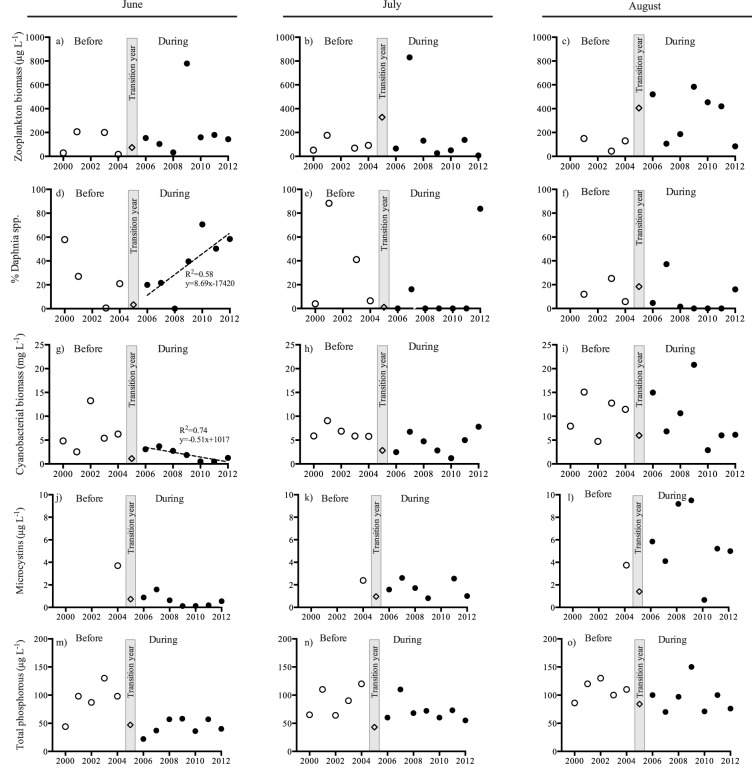
Lake data from Lake Ringsjön (mean + SD) showing total zooplankton biomass (a–c), the proportion of *Daphnia* spp. (%) of total zooplankton biomass (d–f), cyanobacterial biomass (g–i), microcystin concentration (j–l) and total phosphorous concentration (m–o) during June–August before (2000–2004) and during biomanipulation (2006–20012). The first year of biomanipulation (2005) were considered a transition year and were not included in the analysis (gray bar). Regression lines mark significant trends (P<0.05).

In 2005, the first year of biomanipulation, the total catch of cyprinid fish reached around 105 tons (Fig. 2). This was also the year with the highest catch efficiency. Although the total catch has varied among years, the catch efficiency steadily decreased during the period from 2005 to 2008, but thereafter it stabilized at about 800 kg day^−1^ (Fig. 2). This large drop in catchable cyprinid fish suggests that the fish stock, and with that also the predation pressure on zooplankton, decreased with time of biomanipulation. Comparing the effects of the biomanipulation on a monthly basis revealed no significant changes in total zooplankton biomass during any month (Table 1, Fig. 3: a–c). There were no significant trends in the proportion of *Daphnia* spp. before the start of the biomanipulation (Table 1, Fig. 3: d–f). However, in spring (June), the relative contribution of large cladoceran grazers, *Daphnia* spp., increased with time following the biomanipulation (r^2^ = 0.58; P = 0.048; Fig. 3: d), while it remained unaffected in July and August (Table 1, Fig. 3: e–f).

**Table 1:** Results from the linear regressions on total zooplankton biomass, % *Daphnia* spp. (biomass), cyanobacterial biomass, microcystin concentration and total phosphorous concentration in the western basin of Lake Ringsjön before (2000–2004) and during biomanipulation (2006–2012).

	June						July						August					
	Before			During			Before			During			Before			During		
Total phosphorous (unfilt.)	F_(1,3)_	R^2^	P	F_(1,5)_	R^2^	P	F_(1,3)_	R^2^	P	F_(1,5)_	R^2^	P	F_(1,3)_	R^2^	P	F_(1,5)_	R^2^	P
	3.122	0.510	0.175	0.995	0.166	0.364	1.357	0.311	0.328	0.990	0.165	0.365	0.215	0.067	0.675	0.056	0.011	0.822
Σ Zooplankton biomass	F_(1,2)_	R^2^	P	F_(1,5)_	R^2^	P	F_(1,2)_	R^2^	P	F_(1,5)_	R^2^	P	F_(1,1)_	R^2^	P	F_(1,5)_	R^2^	P
	0.006	0.003	0.945	0.029	0.006	0.871	0.017	0.008	0.909	1.158	0.188	0.331	0.153	0.133	0.763	0.124	0.024	0.739
% *Daphnia* spp.	F_(1,2)_	R^2^	P	F_(1,5)_	R^2^	P	F_(1,2)_	R^2^	P	F_(1,5)_	R^2^	P	F_(1,1)_	R^2^	P	F_(1,5)_	R^2^	P
	2.943	0.595	0.228	6.798	0.576	**0.048**	0.080	0.038	0.804	2.072	0.293	0.210	0.015	0.015	0.922	0.281	0.053	0.619
Σ Cyanobacterial biomass	F_(1,3)_	R^2^	P	F_(1,5)_	R^2^	P	F_(1,3)_	R^2^	P	F_(1,5)_	R^2^	P	F_(1,3)_	R^2^	P	F_(1,5)_	R^2^	P
	0.156	0.049	0.719	13.94	0.736	**0.014**	0.506	0.144	0.528	0.461	0.084	0.527	0.104	0.033	0.768	1.235	0.198	0.317
Microcystin concentration	F_(d.f.)_	R^2^	P	F_(1,5)_	R^2^	P	F_(d.f.)_	R^2^	P	F_(1,4)_	R^2^	P	F_(d.f.)_	R^2^	P	F_(1,5)_	R^2^	P
	n/a	n/a	n/a	3.262	0.395	0.131	n/a	n/a	n/a	0.183	0.044	0.691	n/a	n/a	n/a	0.268	0.051	0.627

As only one year of microcystin samples exist prior to the biomanipulation (2004) no analysis could be made on trends in toxins before the biomanipulation (n/a). Significant results (P<0.05) are displayed in bold.

Total cyanobacterial biomass showed a significant decrease in spring (June) with time of biomanipulation (Table 1, Fig. 3: g). Due to this, the cyanobacterial biomass now fluctuates on a considerably lower level in June compared to the period prior to the biomanipulation. These effects were not seen in any of the other summer months investigated (Table 1, Fig. 3: h–i). Although not significant, the cyanobacterial toxins (microcystins) showed similar patterns as the cyanobacterial biomass with much lower concentrations during the biomanipulation compared to before the biomanipulation (Table 1, Fig. 3: j). In July and August, the microcystin concentration still fluctuated around the same level as measured prior to the biomanipulation (2004), while it dropped considerably during June compared to the measured levels in 2004 (Table 1, Fig. 3: j–l). Total phosphorous concentrations in the lake showed no trends, neither before nor during the biomanipulation, with similar concentrations between years and months (Table 1, Fig. 3: m–o).

### Field grazing experiment

**Figure 4:**
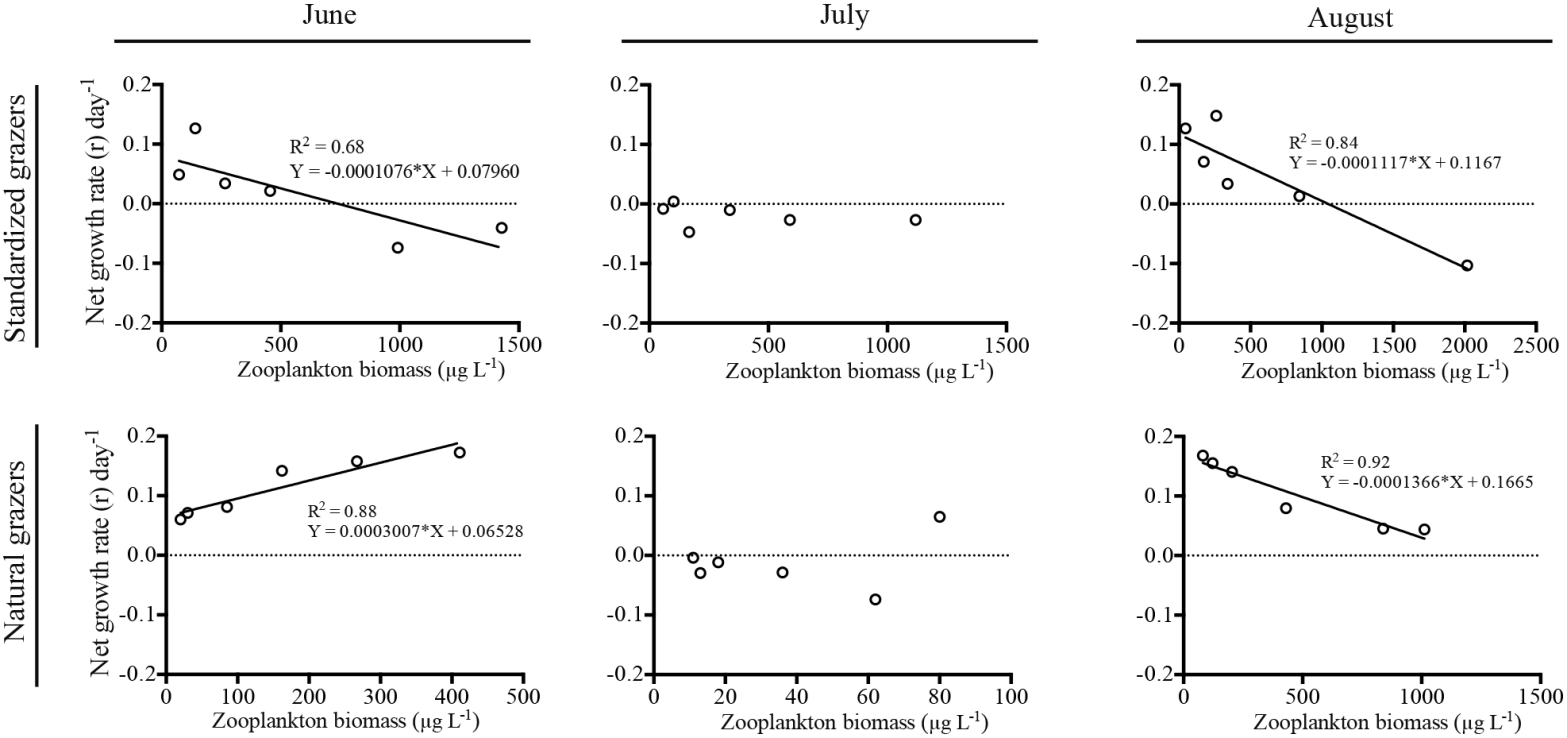
Net growth rates (r) of the cyanobacterial community during June, July and August in relation to grazer abundance. Top panel = *Daphnia* dominated community (standardized grazers) and Lower panel = natural zooplankton community. Regression lines show significant (p<0.05) relations.

Both natural and standardized (large *Daphnia*) zooplankton were, to some extent, able to graze on the cyanobacterial community. The community dominated by *Daphnia magna* was able to strongly affect the cyanobacterial community in both June and August, while the natural community was only able to exert significant grazing in August (Table 2, Fig. 4). However, in the natural community, not even the highest zooplankton density managed to induce a relative algal growth rate (r) below zero, i.e. their grazing rate was lower than the algal growth rate (Fig. 4). Furthermore, in June, the natural grazer community even had a positive effect on cyanobacterial growth, leading to a net increase in cyanobacterial biomass (Fig. 4). None of the two communities had any significant effect on the cyanobacterial community in July (Table 2).

**Table 2:** Results from regressions on cyanobacterial growth rate (r) and relative change in microcystins concentration (r) in relation to zooplankton biomass for both the a) natural zooplankton community and b) the *Daphnia* dominated community.

a)	Natural community								
	June			July			August		
	R^2^	t_(d.f. = 4)_	p	R^2^	t_(d.f. = 4)_	p	R^2^	t_(d.f. = 4)_	p
*Cyanobacteria*	0.881	5.453	**0.005**	0.092	0.638	0.558	0.959	−6.734	**0.003**
*Total microcystins*	0.002	0.094	0.930	0.145	−0.824	0.465	0.563	2.270	0.086
*Extracellular microcystins*	0.125	0.756	0.492	0.189	0.965	0.389	0.001	0.064	0.952
**b)**	***Daphnia*** **-dominated community**								
	**June**			**July**			**August**		
	**R^2^**	**t_(d.f. = 4)_**	**p**	**R^2^**	**t_(d.f. = 4)_**	**p**	**R^2^**	**t_(d.f. = 4)_**	**p**
*Cyanobacteria*	0.822	−2.890	**0.045**	0.102	−0.673	0.538	0.835	−4.502	**0.011**
*Total microcystins*	0.403	−1.644	0.176	0.426	−1.722	0.160	0.239	1.122	0.325
*Extracellular microcystins*	0.812	4.157	**0.014**	0.442	1.779	0.150	0.797	3.963	**0.017**

Significant results (P<0.05) are displayed in bold.

**Figure 5:**
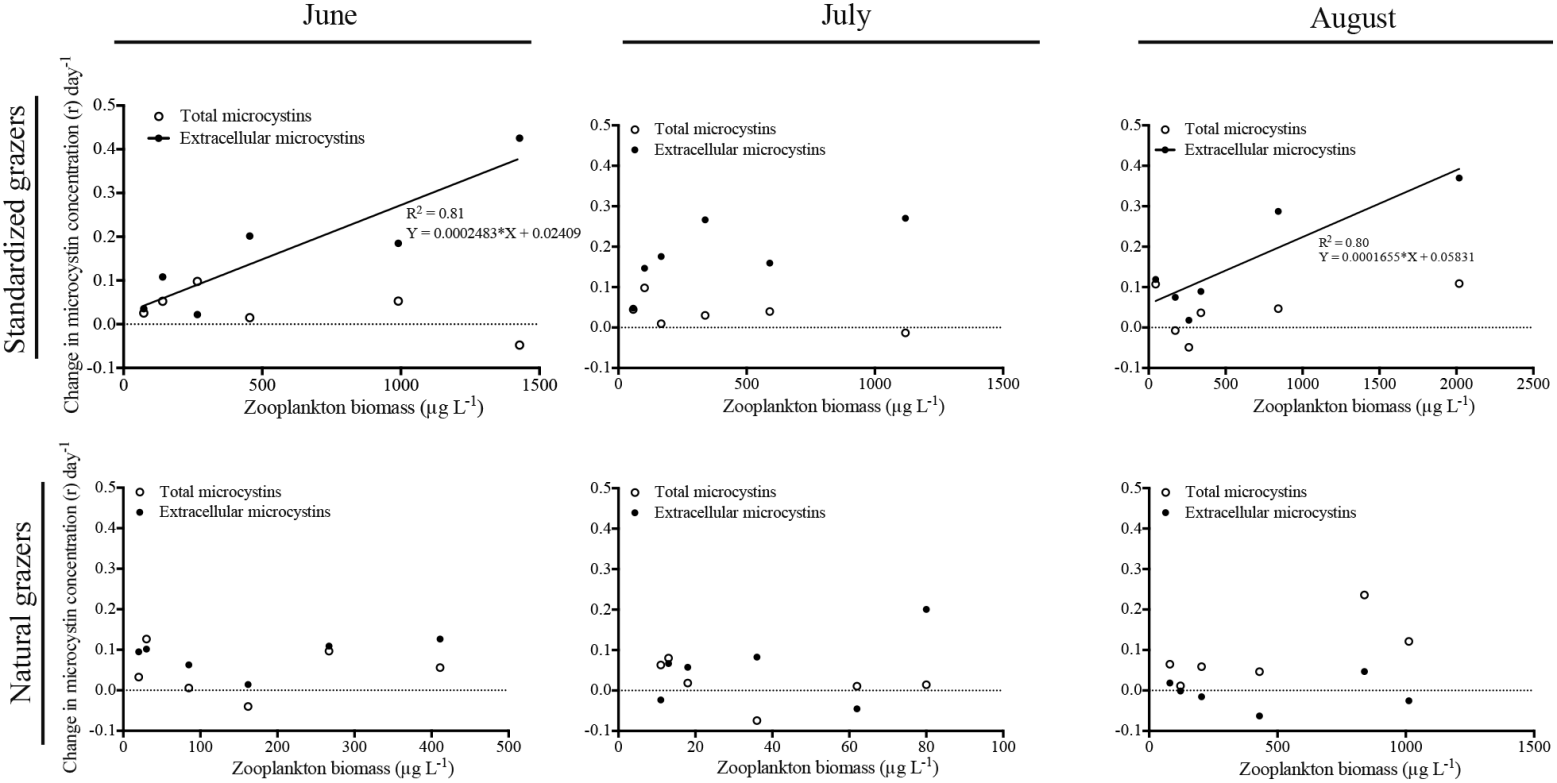
Net change in total microcystin (open circles) and extracellular microcystin concentration (closed circles) in June, July and August in relation to zooplankton biomass. Top panel = *Daphnia* dominated community (standardized grazers) and Lower panel = natural zooplankton community. Regression lines show significant (p<0.05) relations.

Total microcystin concentrations (including both intra- and extracellular microcystins, i.e. inside cells and dissolved in the water) were not affected by zooplankton biomass at any of the study occasions (Table 2). However, during June and August, when the *Daphnia* dominated community was able to exert significant grazing on the cyanobacterial community, there was a significant increase in the extracellular microcystin concentration (Table 2, Fig. 5), indicating that the toxins were transferred from the cyanobacteria to the water. No changes in extracellular concentration were seen in any other case (Table 2, Fig. 5).

## Discussion

Here we show that cascading effects of a whole lake biomanipulation can be tracked from fish removal all the way down to reduced cyanobacterial toxin levels during years of biomanipulation compared to the time before the biomanipulation. However, the effects of biomanipulation in Lake Ringsjön were only visible in spring (June), and although no differences in total zooplankton biomass were found, the change in cyanobacterial biomass and the trends in microcystin concentration coincided with an upswing in the proportion of large cladoceran grazers, *Daphnia* spp.; a group very sensitive to predation by fish [29]. Furthermore, results from the lake monitoring data are strengthened by the field grazing experiment, showing that *Daphnia* are more efficient in feeding on the cyanobacteria than the zooplankton community now present in the lake. Moreover, despite there were no effects on the total microcystin level, the *Daphnia* dominated gradient made a larger pool of the toxins dissolved in the water and thereby available for bacterial degradation [30]. This may be one of the reasons for the observed decreasing trend in lake toxin levels following an upswing in the proportion of *Daphnia* spp. in June. Total phosphorous concentrations did not change significantly with time and can hence not explain the observed gradual decrease in cyanobacterial biomass. However, the levels of total phosphorous were on a lower and more constant level during the years of biomanipulation. One factor that potentially could explain this pattern could be reduced re-suspension of sediment by bentivorous fish (such as bream) [25].

The effects on lower trophic levels following cyprinid fish removal, but the lack of top-down effects on overall zooplankton biomass, are consistent with the findings from previous biomanipulation attempts in Lake Ringsjön [31]. However, here we argue that the observed effects on lower trophic levels in June are likely a result of top-down control through a change in the relative zooplankton composition, with a higher proportion of large cladoceran grazers. That none of the other months investigated showed any trends in *Daphnia* spp. proportion, neither before nor during the biomanipulation, could be due to the increased predation pressure in the lake at that time of the year resulting from the hatching and rapid growth of 0+ fish [32]. Recruitment of 0+ fish, and thereby increased predation pressure on zooplankton, is generally found to increase even further following biomanipulation [33], something that was also seen in Lake Ringsjön during the first biomanipulation attempt during the 1990s [34]. Another factor contributing to the collapse of the *Daphnia* spp. in July could be changes in phytoplankton species composition due to natural succession in lakes [35]. Succession towards larger sized cyanobacterial species as the growth season progresses could potentially lead to clogging of the feeding apparatus of the daphnids [24], [36]. The combination of these factors would render large zooplankton sandwiched between increased predation pressure and poor food quality, possibly contributing to the low biomass of large cladoceran grazers observed in July and August and, with that, the lack of a trophic cascade following biomanipulation during these months. In addition, although *Daphnia* previously adapted to toxic cyanobacteria have been shown to be able to suppress phytoplankton biomass at high microcystin concentration [37], the higher microcystin levels in July and August, compared to in June, may also have contributed to the reduced performance of the herbivores leading to their population decline [38], [39].

The field grazing experiment clearly showed that large *Daphnia* were more efficient in grazing on cyanobacteria than the natural grazer community. While the *Daphnia* strongly affected the cyanobacterial biomass negatively both in June and August, the natural community was only able to affect them negatively in August. In fact, *Daphnia* stocking has previously successfully been used as a restoration tool in combination with piscivorous fish stocking in biomanipulation attempts to control algal blooms [18]. Furthermore, although the natural community was able to exert significant grazing on the cyanobacterial community in August, they were not even at the highest abundances able to reduce the growth rate of the cyanobacteria below zero, i.e. they were not even able to compensate for the cyanobacterial growth during the 72 hour experiment. The fact the natural community were not able to exert significant grazing in June, when it contained a larger proportion of large cladoceran grazers, can be explained by that the biomass of zooplankton found in the lake was considerably lower in June than in August, thereby generating a lower grazing pressure on the cyanobacteria. *Daphnia*, however, generally managed to push the net growth rate of the cyanobacteria below zero i.e. their grazing did not only compensate for cyanobacterial growth during the experiment, but also affected the standing biomass. In June, an increase in biomass of the natural zooplankton community boosted a significant growth of cyanobacteria. A likely explanation is that the natural community then mainly consisted of copepods and small cladocerans, such as *Bosmina* and *Chydorus*, and that these small cladoceran species were not able to graze on the relatively large cyanobacteria. This is in line with the findings by Dawidowicz (1990) [24] who showed that the existing lake community in the moderately eutrophic Lake Ros (Northern Poland) was only able to graze on the smaller sized phytoplankton species. In July, none of the grazer gradients showed significant grazing on the cyanobacterial community. At this point the cyanobacterial community was dominated by large filaments of *Anabaena crassa* and although *Daphnia magna* has been shown to be able to feed on large sized phytoplankton [40], the filamentous character of the *Anabaena crassa* might have led to clogging of the feeding apparatus [41]–[43].

The cyanotoxin (microcystin) levels remained unaffected by grazers throughout the grazing experiment. However, the *Daphnia* dominated gradient induced an increased concentration of microcystins dissolved in the water, i.e. the extracellular fractions of microcystins. This effect was never observed for the natural grazer community, not even when their grazing rate significantly reduced the cyanobacterial growth in August. *Daphnia*, being a less selective grazer [44], likely ingest more of the toxic cyanobacteria than the natural community, which might feed more selectively, or be too small to feed on large cyanobacteria. If so, the excretion of damaged cells by *Daphnia* is the likely reason for the higher levels of extracellular microcystins in the water. Furthermore, Jang et al. (2007) [45] have suggested that cyanobacteria actively release microcystins into the water as a response to zooplankton. Irrespective of the mechanism behind the increased extracellular toxin level, the toxins are made available for bacterial- and photochemical degradation by large *Daphnia* grazers, but not by the smaller herbivores [30], [46]. Although higher dissolved concentrations of cyanotoxins will have an immediate negative effect for ecosystem services, such as drinking water and recreation, it will expose the toxins to degradation and thereby lead to improved water quality in the long run. Furthermore, given that microcystins are not broken down internally in the cyanobacterial cells [47], the breakdown processes of extracellular toxins would likely be the major route for reducing the concentration of microcystins in the water. Hence, a higher portion of large herbivores may not only reduce the abundance of nuisance cyanobacteria, but also reduce the toxin levels, thereby fulfilling one of the main goals with biomanipulation, a notion strengthened by our lake monitoring data. Although our dataset on microcystins only includes one year prior to the biomanipulation it is clear that the levels of microcystins in June during that year were much higher than during the years with biomanipulation - when we see a tendency of reduced toxin concentration with time of biomanipulation. During both July and August the levels of microcystins during years of biomanipulation is still at the same level as they were before the biomanipulation, following the patterns in cyanobacterial biomass. This strongly suggests that the biomanipulation, through top down control, has led to a reduction in cyanobacterial toxin levels during early summer. As shown in our field grazing experiment, *Daphnia* grazing did not lead to a drop in total toxin concentration in the water but rather a relative change in the fraction of extracellular vs. intracellular toxins. This could potentially explain the results from the lake as we, as in the field grazing experiment, see a reduction in cyanobacterial biomass but only tendencies to reduced toxin levels as the lake monitoring samples do not discriminate between the different fractions of microcystins.

In conclusion, both monitoring- and experimental data show that biomanipulations, through top-down mediated trophic cascades, can lead to improved water quality expressed both as reduced cyanobacterial biomasses and lowered toxin levels in spring. Following cyprinid fish removal and reduced predation pressure on zooplankton, *Daphnia* spp. became more dominant leading to more efficient top down control on phytoplankton. This led to a reduction in the pool of toxin producing cyanobacteria and microcystins found in June. Furthermore, the grazing experiment revealed that an increased abundance of *Daphnia* leads to higher levels of extracellular toxins making them more susceptible to degradation that would further improve water quality. None of these cascading effects, which ultimately lead to better water quality, were seen when the zooplankton community was dominated by smaller species, neither in the field nor in the grazing experiment. Hence, we here show the importance of fish removal as a tool for top-down mediated cascading effects leading all the way to reduced toxin levels. If the spring clear water phase can be prolonged, as our data suggest, with increased abundance of large and efficient grazers further into the summer season, this would reduce the amount of phytoplankton through bottom-up mediated effects. This would ultimately reduce the formation of nuisance and toxic blooms even further into the season which would improve not only the ecological status of the lake, but also make it more valuable as a resource for ecosystem services, such as recreation and drinking water supply.

## Supporting Information

Data S1 **Experimental data.**
(PDF)
