# Supplementary material for: Linking Cascading Effects of Fish Predation and Zooplankton Grazing to Reduced Cyanobacterial Biomass and Toxin Levels Following Biomanipulation
Source: PLoS One. 2014 Nov 19;9(11):e112956. doi: 10.1371/journal.pone.0112956 (PMC4237340; doi:10.1371/journal.pone.0112956)
Supplement: Data S1 — Experimental data. (PDF) [file pone.0112956.s001.pdf]

Experimental data

Linking cascading effects of fish predation and zooplankton grazing to reduced cyanobacterial biomass and toxin levels following biomanipulation

Ekvall et al. 2014

June

Gradient	Total Zoopl.	Large cladocerans	Small cladocerans	Copepods	Cyano. biomass T0	Cyano. biomass T1	Total Microcystin T0	Total Microcystin T1	Extracell. Microcystins T0	Extracell. Microcystins T1	Calculated r day^{-1}		
	ug/L	ug/L	ug/L	ug/L	mg/L	mg/L	ug/L	ug/L	ug/L	ug/L	Cyanobacteria	Total Microcystin	Extracell. Microcystins
NG 0.25	19.5	2.8	1.1	15.6	1.852	2.218	0.844	0.932	0.100	0.133	0.060	0.033	0.095
NG 0.5	30.2	4.8	3.8	21.6	2.171	2.688	0.830	1.213	0.126	0.171	0.071	0.126	0.102
NG 1	85.4	15.9	4.9	64.7	1.408	1.796	1.062	1.080	0.135	0.163	0.081	0.006	0.063
NG 2	162.3	37.0	12.4	112.9	1.390	2.127	1.179	1.046	0.162	0.169	0.142	-0.040	0.014
NG 4	266.5	56.3	20.9	189.4	1.792	2.878	0.973	1.301	0.129	0.179	0.158	0.097	0.109
NG 6	410.8	88.7	32.0	290.1	1.750	2.938	1.317	1.557	0.104	0.152	0.173	0.056	0.126
SG 0.25	73.1	70.8	0.1	2.2	1.629	1.887	1.022	1.104	0.116	0.129	0.049	0.026	0.035
SG 0.5	141.5	137.5	0.1	4.0	1.199	1.752	0.766	0.897	0.115	0.159	0.126	0.052	0.108
SG 1	266.0	262.5	0.2	3.4	1.385	1.534	0.916	1.228	0.117	0.125	0.034	0.098	0.022
SG 2	455.1	454.1	0.3	0.7	1.315	1.404	0.919	0.962	0.106	0.194	0.022	0.015	0.201
SG 4	990.6	987.4	0.6	2.6	1.626	1.303	0.842	0.987	0.116	0.202	-0.074	0.053	0.185
SG 6	1427.6	1424.8	0.3	2.4	1.524	1.349	0.951	0.824	0.125	0.448	-0.041	-0.048	0.425

July

Gradient	Total Zoopl.	Large cladocerans	Small cladocerans	Copepods	Cyano. biomass T0	Cyano. biomass T1	Total Microcystin T0	Total Microcystin T1	Extracell. Microcystins T0	Extracell. Microcystins T1	Calculated r day^{-1}		
	ug/L	ug/L	ug/L	ug/L	mg/L	mg/L	ug/L	ug/L	ug/L	ug/L	Cyanobacteria	Total Microcystin	Extracell. Microcystins
NG 0.25	10.6	0.0	4.8	5.8	12.158	12.014	1.464	1.768	0.236	0.220	-0.004	0.063	-0.023
NG 0.5	12.5	0.0	4.6	7.9	13.595	12.439	1.418	1.804	0.140	0.171	-0.030	0.080	0.067
NG 1	17.9	0.5	9.3	8.1	10.755	10.385	1.757	1.857	0.160	0.190	-0.012	0.018	0.057
NG 2	36.1	1.0	20.9	14.3	11.551	10.604	2.221	1.778	0.181	0.232	-0.028	-0.074	0.083
NG 4	62.0	1.4	38.3	22.3	12.891	10.327	1.920	1.981	0.173	0.151	-0.074	0.011	-0.045
NG 6	79.7	2.3	44.4	33.0	9.949	12.088	2.245	2.342	0.148	0.270	0.065	0.014	0.200
SG 0.25	58.2	52.3	0.8	5.1	11.265	10.989	2.032	2.325	0.156	0.180	-0.008	0.045	0.048
SG 0.5	101.6	95.3	2.0	4.3	11.419	11.562	1.897	2.548	0.141	0.219	0.004	0.098	0.147
SG 1	167.2	160.5	2.4	4.2	11.052	9.589	1.974	2.032	0.154	0.261	-0.047	0.010	0.176
SG 2	337.6	332.2	2.0	3.5	11.380	11.043	1.780	1.948	0.174	0.387	-0.010	0.030	0.266
SG 4	589.8	585.6	2.3	2.0	10.551	9.730	1.820	2.050	0.145	0.234	-0.027	0.040	0.160
SG 6	1119.0	1113.5	2.4	3.1	8.885	8.196	2.253	2.166	0.219	0.493	-0.027	-0.013	0.270

August

Gradient	Total Zoopl.	Large cladocerans	Small cladocerans	Copepods	Cyano. biomass T0	Cyano. biomass T1	Total Microcystin T0	Total Microcystin T1	Extracell. Microcystins T0	Extracell. Microcystins T1	Calculated r day^{-1}		
	ug/L	ug/L	ug/L	ug/L	mg/L	mg/L	ug/L	ug/L	ug/L	ug/L	Cyanobacteria	Total Microcystin	Extracell. Microcystins
NG 0.25	81.0	2.5	32.5	46.0	10.245	16.945	2.699	3.280	0.209	0.221	0.168	0.065	0.019
NG 0.5	121.8	6.5	54.1	61.2	9.481	15.103	2.200	2.275	0.225	0.224	0.155	0.011	-0.001
NG 1	203.9	16.2	88.0	99.7	9.233	14.075	1.988	2.371	0.199	0.190	0.141	0.059	-0.015
NG 2	429.6	38.1	204.5	187.0	9.795	12.436	2.647	3.044	0.225	0.186	0.080	0.047	-0.063
NG 4	838.7	86.2	393.5	358.9	11.000	12.597	2.019	4.099	0.246	0.283	0.045	0.236	0.047
NG 6	1012.2	111.1	461.7	439.4	12.640	14.422	2.628	3.779	0.245	0.227	0.044	0.121	-0.025
SG 0.25	45.4	25.8	6.7	12.9	9.846	14.400	2.378	3.281	0.219	0.313	0.127	0.107	0.119
SG 0.5	173.4	149.1	10.7	13.6	12.852	15.888	4.793	4.688	0.238	0.298	0.071	-0.007	0.075
SG 1	261.2	233.5	11.4	16.2	10.267	16.008	4.420	3.818	0.204	0.215	0.148	-0.049	0.018
SG 2	338.7	310.3	13.4	15.0	10.486	11.606	3.403	3.799	0.179	0.234	0.034	0.037	0.089
SG 4	841.6	817.9	9.3	14.5	9.286	9.656	4.066	4.679	0.190	0.450	0.013	0.047	0.287
SG 6	2017.5	1994.1	8.3	15.1	10.025	7.359	3.368	4.669	0.192	0.582	-0.103	0.109	0.370
